# FGFR2 is overexpressed in myxoid liposarcoma and inhibition of FGFR signaling impairs tumor growth *in vitro*

**DOI:** 10.18632/oncotarget.4046

**Published:** 2015-05-08

**Authors:** Helen Künstlinger, Jana Fassunke, Hans-Ulrich Schildhaus, Benedikt Brors, Carina Heydt, Michaela Angelika Ihle, Gunhild Mechtersheimer, Eva Wardelmann, Reinhard Büttner, Sabine Merkelbach-Bruse

**Affiliations:** ^1^ Institute of Pathology, University Hospital Cologne, Cologne, Germany; ^2^ Institute of Pathology, University Hospital Göttingen, Göttingen, Germany; ^3^ Computational Oncology, German Cancer Research Center (DKFZ), Heidelberg, Germany; ^4^ Institute of Pathology, University Hospital Heidelberg, Heidelberg, Germany; ^5^ Gerhard-Domagk-Institute of Pathology, University Hospital Münster, Münster, Germany

**Keywords:** myxoid liposarcoma, whole-genome microarray, gene expression, fibroblast growth factor, FGFR inhibition

## Abstract

Myxoid liposarcomas account for more than one third of liposarcomas and about 10% of all adult soft tissue sarcomas. The tumors are characterized by specific chromosomal translocations leading to the chimeric oncogenes *FUS-DDIT3* or *EWS1R-DDIT3*. The encoded fusion proteins act as aberrant transcription factors. Therefore, we implemented comparative expression analyses using whole-genome microarrays in tumor and fat tissue samples. We aimed at identifying differentially expressed genes which may serve as diagnostic or prognostic biomarkers or as therapeutic targets. Microarray analyses revealed overexpression of *FGFR2* and other members of the FGF/FGFR family. Overexpression of FGFR2 was validated by qPCR, immunohistochemistry and western blot analysis in primary tumor samples. Treatment of the myxoid liposarcoma cell lines MLS 402 and MLS 1765 with the FGFR inhibitors PD173074, TKI258 (dovitinib) and BGJ398 as well as specific siRNAs reduced cell proliferation, induced apoptosis and delayed cell migration. Combination of FGFR inhibitors with trabectedin further increased the effect. Our study demonstrates overexpression of FGFR2 and a functional role of FGFR signaling in myxoid liposarcoma. As FGFR inhibition showed effects on proliferation and cell migration and induced apoptosis *in vitro*, our data indicate the potential use of FGFR inhibitors as a targeted therapy for these tumors.

## INTRODUCTION

Liposarcomas are rare malignant tumors that preferentially occur in deep soft tissue. However, representing 10-15% of all soft tissue sarcomas they are one of the most frequent sarcoma subtypes [1]. Based on histological and cytogenetic properties, liposarcomas can be distinguished into three different entities, i.e. well-differentiated/dedifferentiated (WD/DDLPS), myxoid/round cell (MRLPS) as well as pleomorphic liposarcomas (PLPS) [2, 3]. WD/DDLPS are genetically defined by giant marker or ring chromosomes with an amplification on chromosome 12 affecting among others the genes *MDM2* and *CDK4* [4]. Likewise, myxoid and round cell liposarcomas are considered as a common tumor entity which is characterized by a reciprocal translocation of the *DDIT3* gene with either *FUS* (>90%) or *EWS1R* [5-8]. The translocations t(12;16)(q13;p11) and accordingly t(12;22)(q13;p12) are specific for this tumor entity and absent in other myxoid look-alikes such as myxofibrosarcoma [9]. The translocation leads to the fusion of the involved genes and the formation of a chimeric protein.

Besides the initial translocation only little is known about tumorigenic pathways deregulated by the chimeric protein [10]. As the *DDIT3* fusion proteins are most likely to act as aberrant transcription factors, the transcriptional control of many genes may be altered. Such differentially expressed genes could be diagnostic or prognostic biomarkers as well as therapeutic targets. In order to identify multiple differentially expressed genes at the same time, cDNA microarrays are particularly suitable. They have already been performed in different sarcoma entities and revealed subtype specific expression signatures as well as basis for novel therapeutic approaches [11-15]. Nevertheless, the identification of candidate target structures based on gene expression profiling alone does not provide reliable evidence for the implementation of new therapeutic strategies. Rather, results need to be carefully validated and functional studies have to confirm the suitability of identified candidates.

Treatment of liposarcomas with the new chemotherapeutic compound trabectedin (ET-743) revealed promising results [16, 17], but molecularly targeted therapies are not yet available. For other soft tissue sarcomas some success with targeted therapies has been achieved in specific subtypes, but their implementation remains far behind treatment regimes in carcinomas [18, 19, 20]. An interesting family of receptor tyrosine kinases that can successfully be targeted and whose role in tumorigenesis has been revealed for different sarcoma entities are fibroblast growth factor receptors (FGFRs). FGFR1 amplification has been described in osteosarcomas and rhabdomyosarcomas were identified to carry activating mutations in *FGFR4* [21, 22]. Furthermore, activation of FGFR signaling through amplification of the adaptor *FRS2* has recently been described in high-grade liposarcomas [23].

The implementation of therapies with defined molecular targets requires the identification of new key molecules. Therefore, we implemented comparative whole-genome microarray analyses in primary myxoid liposarcomas and fat tissue samples. FGFR2, together with other members of the FGF/FGFR family, showed overexpression. FGFR2 expression was further analyzed in primary tumors and myxoid liposarcoma cell lines were treated with FGFR silencing siRNAs and tyrosine kinase inhibitors. Inhibitors were additionally combined with trabectedin. Our study investigates a potential role of FGFR signaling in myxoid liposarcomas and the use of FGFR inhibitors as a novel targeted treatment approach for these tumors.

## RESULTS

### Microarray analyses

In order to identify new key molecules in the pathogenesis of myxoid liposarcoma, whole-genome microarray analyses were performed with seven tumor samples and an RNA pool of eight normal fat tissue samples with excellent RNA quality (RNA integrity number (RIN) value ≥ 7.0, Table 1 and Supplemental Figure S1 A).

**Table 1:** Cohort of tumor and fat control samples Samples with RIN values printed in bold were used for microarray analysis.

Tumor Samples	Case	Cryo	FFPE	Rearrangement	RIN Cryo	RIN FFPE
	MLS 1	X	X	FUS/DDIT3	**8.6**	2.4
	MLS 2	X	X	FUS/DDIT3	2.8	2.4
	MLS 3	X	X	FUS/DDIT3	3	2.4
	MLS 4	X	X	FUS	4.1	2.4
	MLS 5	X	X	FUS	5.8	1.8
	MLS 6		X	FUS		2.9
	MLS 7		X	EWS1R/DDIT3		2.3
	MLS 8		X	FUS/DDIT3		2.4
	MLS 9		X	FUS/DDIT3		2.5
	MLS 10		X	FUS/DDIT3		2.5
	MLS 11	X		DDIT3	**9.3**	
	MLS 12	X		FUS/DDIT3	**9.4**	
	MLS 13	X		FUS/DDIT3	**7.9**	
	MLS 14	X		FUS/DDIT3	**9.4**	
	MLS 15	X		FUS	**9.7**	
	MLS 16	X		FUS/DDIT3	**7.6**	
**Fat Tissue**	**Case**	**Cryo**	**FFPE**	**Localization**	**RIN Cryo**	**RIN FFPE**
	FAT 1	X		Pericolic	6.7	
	FAT 2	X		Pericolic	5.4	
	FAT 3	X		Retroperitoneal/perirenal	**7.9**	
	FAT 4	X		Pericolic	6.4	
	FAT 5	X		Pericolic	**7.1**	
	FAT 6	X		Pericolic	**7.7**	
	FAT 7	X		Pericolic	**7.4**	
	FAT 8	X		Mamma	**8.0**	
	FAT 9	X		Mamma	**7.4**	
	FAT 10	X		Pericolic	**7.0**	
	FAT 11	X		Mamma	6.9	
	FAT 12	X		Pelvine lymph nodes	**7.5**	
	FAT 13	X		Pelvine lymph nodes	6.4	
	FAT 14	X		Pericolic	6.4	
	FAT 15	X		Mamma	5.7	

Cryo: cryo-conserved tissue; FFPE: formalin-fixed, paraffin-embedded tissue; RIN: RNA integrity number; MLS: myxoid liposarcoma.

**Figure 1:**
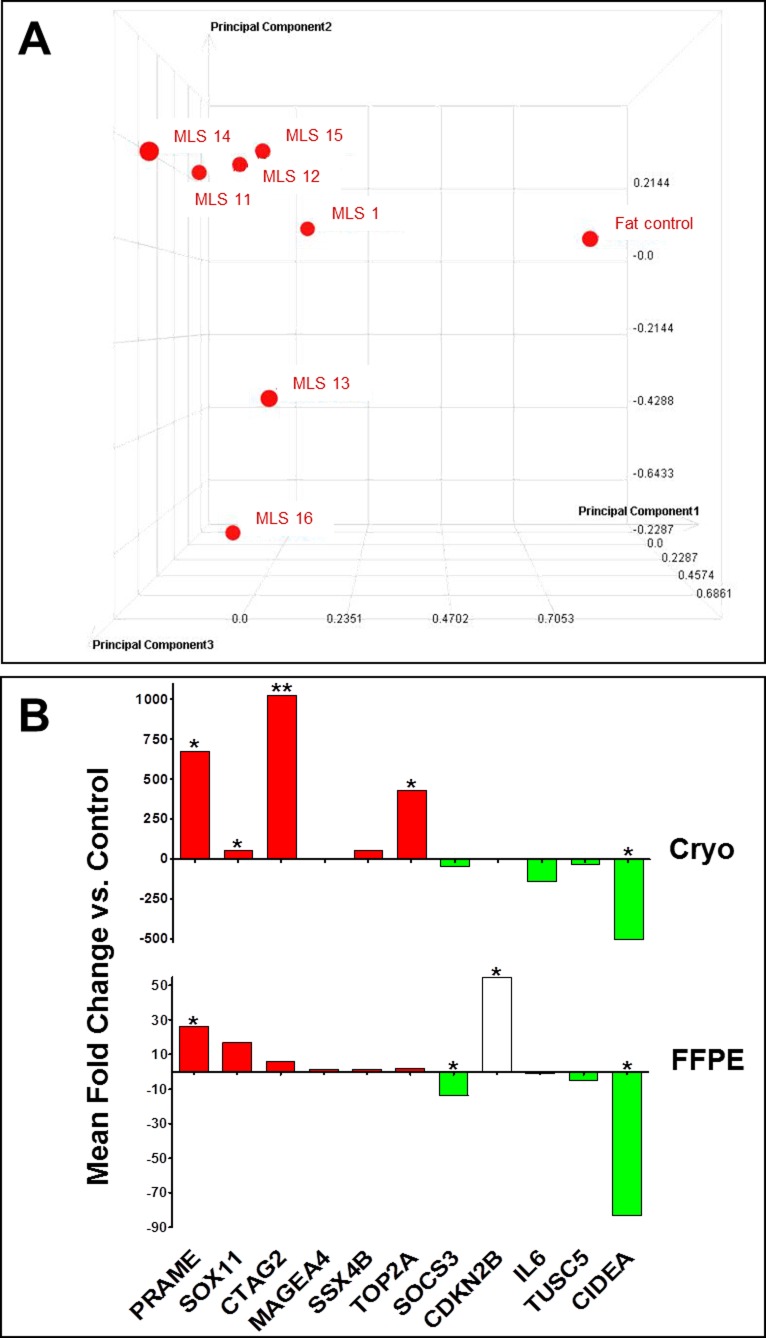
Whole-genome microarray analysis and validation **A.** Three dimensional principal component analysis (PCA) of the gene expression profiles. On X, Y and Z axis arbitrary units for the three different principal components are indicated. Each profile is condensed to a single data point in the three dimensional PCA. The data points' size and distance reflect the three dimensional position within the grid and thus indicate the similarity among the different profiles. **B.** qPCR validation of selected candidate genes in cryo-conserved (top) and FFPE (bottom) primary tumor samples. Red or green colored bars represent genes with confirmed up- or down-regulation (same expression changes as discovered by microarray analysis previously). Data are represented as analysis output of REST [48] (mean fold change of triplicate measurements). *: *p* ≤ 0.05; **: *p* ≤ 0.01; ***: *p* ≤ 0.001.

A principal component analysis (PCA) was carried out to analyze the relative similarity between the expression profiles. The PCA plot (Figure 1A) illustrates a clear difference between the fat control pool and the tumor samples, which show a high degree of similarity among each other. Due to this similarity the tumor samples were compared to the control as a whole. By means of this comparison a multitude of differentially expressed genes could be identified. We detected 7,946 genes with significant twofold or more up- or down-regulation (*p*-value < 0.01). Maximal measured fold changes were ±100. In the range of +80 to +100 and −80 to −100 were 26 genes, respectively. A heat map reflecting the differential expression of several genes in MLS tumor samples compared to normal fat tissue is given in Supplemental Figure S1B.

All MLS samples included in the study were proven to carry a *DDIT3* translocation involving either *FUS* or *EWS1R* using FISH analysis (Supplemental Figure S1C). Additionally, in samples used for microarray analysis and MLS cell lines the exact type of *DDIT3* translocation was determined by RT-PCR and sequencing (Supplemental Table S1). In two tumor samples our analysis revealed so far undescribed variants. Case MLS 12 carries both fusion transcripts of type I and type III. For MLS 1 a variant of type I lacking the last 24 codons of *FUS* exon 7 was identified. A correlation between the differential expression of genes and the particular type of fusion transcript was not detected.

### Microarray validation and identification of suitable reference genes

We analyzed the expression stability of 16 candidate reference genes by means of qPCR using TaqMan^®^ Array Human Endogenous Control Panels in cryo-conserved and formalin-fixed tumor tissue as well as in cryo-conserved fat tissue samples. Data were analyzed using GeNorm and NormFinder software tools, which both detected comparable results. Expression stabilities generated with GeNorm are shown in Supplemental Figure S2. Suitable reference genes should exhibit stability values below 1.5, which was seen in cryo-conserved tissue samples for all genes apart from *PGK1*. In formalin-fixed tissue, stability values for the candidate reference genes turned out to be much higher. As the qPCR validation was also performed in a cohort of formalin-fixed samples, these data were considered preferentially for selection of reference genes. *IPO8* was among the two most stable reference genes in all three tissue types and was therefore selected as one reference gene. *B2M* was chosen as the second reference gene due to its stability in formalin-fixed as well as in cryo-conserved tissue samples. The evaluation of expression data with NormFinder revealed likewise stability values below 1.5 for *IPO8* and *B2M* in all three tissue types.

Results of microarray analyses were validated for eleven genes in the whole tumor group by SYBR Green qPCR. The analyzed genes were selected from those genes with at least twenty fold significantly differential expression (Table 2). RNA pools of cryo-conserved or post-fixed fat tissue were used as controls. Data obtained by qPCR experiments were evaluated using REST (Relative Expression Software Tool) and graphically visualized using GraphPad Prism (Figure 1B). Validation in cryo-conserved tumor samples revealed the same differential expression of all tested genes as measured with microarray analysis. In formalin-fixed tumor tissue ten of eleven genes showed the same expression changes as discovered by microarray analyses previously. Thus, results of microarray analyses could be reproduced by qPCR.

**Table 2:** Candidate genes for qPCR validation Differential expression of selected candidate genes as detected with whole-genome microarrays. Genes in the upper section were up-regulated whereas genes in the bottom section were down-regulated in MLS tumor samples compared to fat tissue as control.

Gene Symbol	Sequence Description	Log (Ratio)	Fold Change	P-value
*PRAME*	Homo sapiens preferentially expressed antigen in melanoma. transcript variant 5. mRNA [NM_206956]	2	100	0
*SOX11*	Homo sapiens SRY (sex determining region Y)-box 11. mRNA [NM_003108]	2	100	0
*CTAG2*	Homo sapiens cancer/testis antigen 2. transcript variant 2. mRNA [NM_020994]	2	100	0
*MAGEA4*	Homo sapiens melanoma antigen family A. 4. transcript variant 2. mRNA [NM_002362]	1.99519	98.89771	0
*SSX4B*	Homo sapiens synovial sarcoma. X breakpoint 4B. transcript variant 1. mRNA [NM_001034832]	1.65495	45.18048	1.55E-07
*TOP2A*	Homo sapiens topoisomerase (DNA) II alpha 170kDa. mRNA [NM_001067]	1.45462	28.48537	1.51E-19
*SOCS3*	Homo sapiens suppressor of cytokine signaling 3. mRNA [NM_003955]	−1.36976	−23.42935	6.78E-39
*CDKN2B*	Homo sapiens cyclin-dependent kinase inhibitor 2B (p15. inhibits CDK4). transcript variant 2. mRNA [NM_078487]	−1.59644	−39.48581	1.90E-23
*IL6*	Homo sapiens interleukin 6 (interferon. beta 2). mRNA [NM_000600]	−1.92363	−83.87525	0
*TUSC5*	Homo sapiens tumor suppressor candidate 5. mRNA [NM_172367]	−1.94155	−87.40817	1.67E-35
*CIDEA*	Homo sapiens cell death-inducing DFFA-like effector a. transcript variant 2. mRNA [NM_198289]	−2	−100	0

### FGFR expression in myxoid liposarcoma

**Figure 2:**
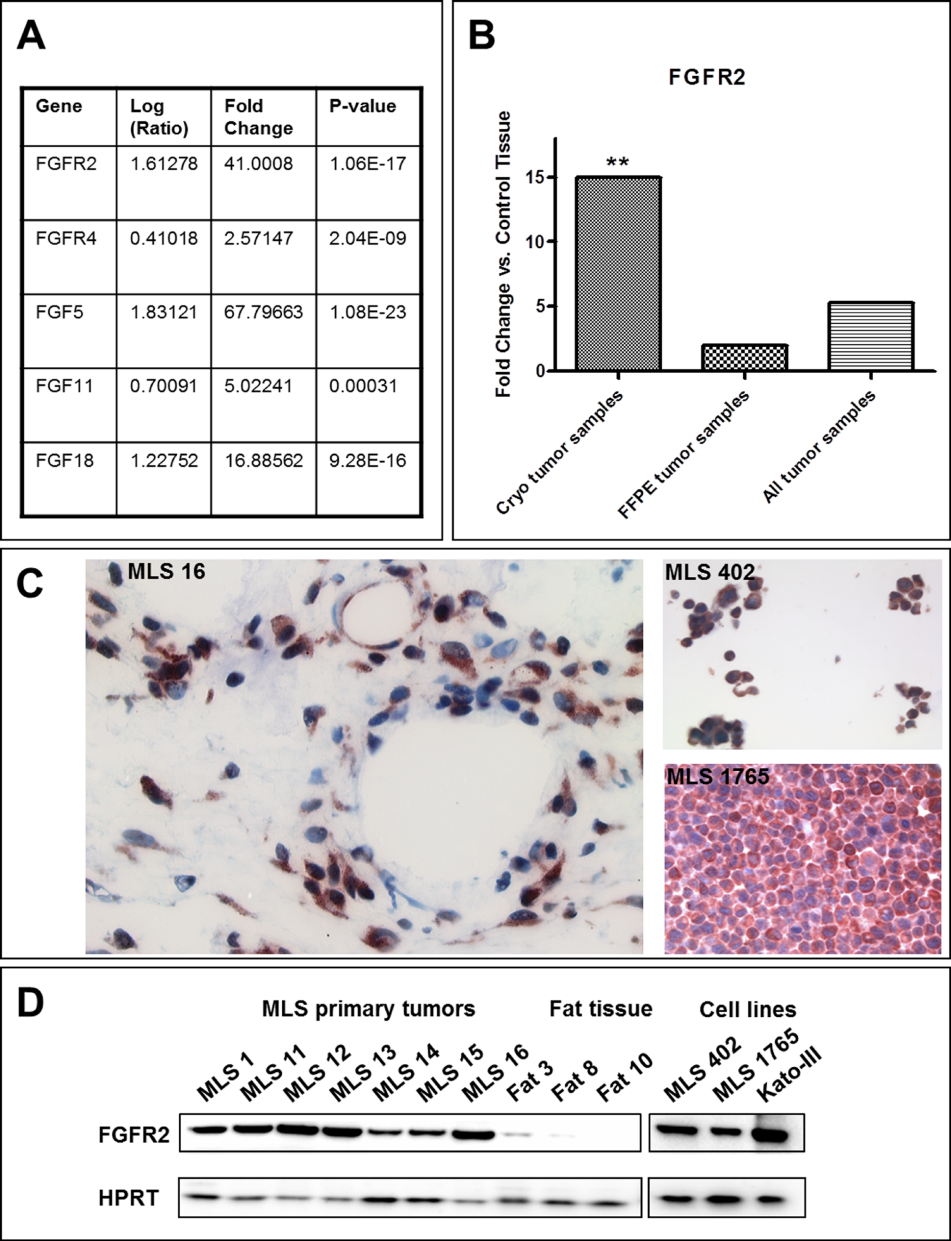
FGFR expression **A.** Differential expression of members of the FGF/FGFR family as detected with the microarray analysis. **B.** Differential expression of *FGFR2* in myxoid liposarcomas detected with qPCR in the whole tumor cohort. Data are represented as analysis output of REST [48] (mean fold change of triplicate measurements). *: *p* ≤ 0.05; **: *p* ≤ 0.01; ***: *p* ≤ 0.001. **C**. FGFR2 immunohistochemistry in MLS primary tumor tissue (left) and cell lines (right). **D**. FGFR2 protein expression in cryo-conserved tumor and fat tissue samples as well as in MLS cell lines detected by western blot analysis. Kato-III cells served as positive control for FGFR2 expression.

By evaluating the microarray analyses multiple genes expressed at significantly different levels were identified. One of these genes was *FGFR2*, which was highly overexpressed in myxoid liposarcoma. In addition to *FGFR2* also other members of the FGF/FGFR family showed an overexpression in the microarray analyses (Figure 2A) further reinforcing the potential role of FGFR signaling in myxoid liposarcomas. In qPCR analyses a clear up-regulation of *FGFR2* gene expression was detected in cryo-conserved as well as in formalin-fixed tumor samples (Figure 2B) confirming the overexpression identified by microarray analyses in the whole tumor cohort.

FGFR2 expression in primary myxoid liposarcomas was also shown on protein level by immunohistochemistry (Figure 2C) and western blot analysis (Figure 2D). Vascular endothelial cells as well as the tumor cells were positive for FGFR2. FGFR2 protein expression was also present in the myxoid liposarcoma cell lines MLS 402 and MLS 1765 (Figures 2C and 2D). In contrast, fat tissue samples showed only weak or no FGFR2 protein expression (Figure 2D).

### FGFR silencing with specific siRNAs

**Figure 3:**
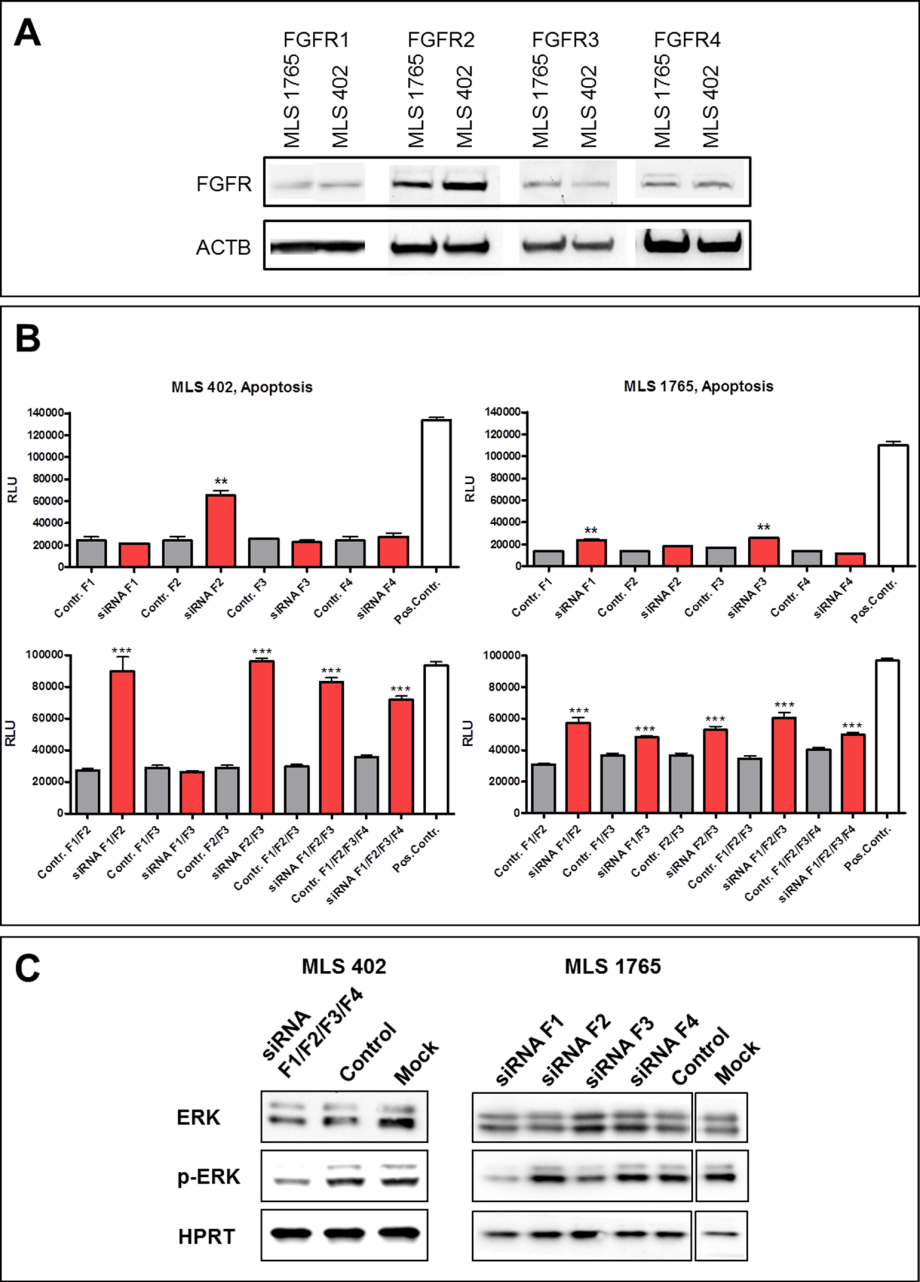
Effects of FGFR siRNA silencing **A.** FGFR1, FGFR2, FGFR3 and FGFR4 protein expression in MLS cell lines detected by western blot analysis. **B.** Apoptosis induction upon FGFR siRNA silencing in MLS cell lines, either of the different receptors alone (top) or combined knock-down of several FGFRs (bottom). As negative control cells were transfected with Stealth RNAi™ Negative Control Duplexes with the corresponding GC content (Contr. F1, F2, F3, F4). AllStars Hs Cell Death siRNA served as transfection control and as positive control for apoptosis induction (Pos.Contr.). Data of quintuplicates are represented as mean +/− SEM. *: *p* ≤ 0.05; **: *p* ≤ 0.01; ***: *p* ≤ 0.001. F1:FGFR1; F2:FGFR2; F3: FGFR3; F4:FGFR4. **C.** Effects of FGFR silencing on downstream signaling in MLS cell lines 48 h after transfection with specific siRNAs. As negative control cells were transfected with Stealth RNAi™ Negative Control Duplexes with the corresponding GC content.

In order to analyze the functional role of FGFR signaling in the myxoid liposarcoma cell lines MLS 402 and MLS 1765, cells were transiently transfected with specific siRNAs. Silencing efficiency of the used siRNAs was shown by qPCR 48 h after transfection (Supplemental Figure S3). As both cell lines express all four FGFRs (Figure 3A), the receptors were knocked-down separately as well as in combination. Figure 3B shows apoptosis induction upon FGFR silencing, either of FGFR1, 2, 3 and 4 alone or by combined knock-down of several receptors. In MLS 402 single receptor silencing of FGFR2 induced apoptosis, whereas knock-down of the other receptors alone showed no effect (Figure 3B, left upper graph). Accordingly, the combined knock-down of more than one FGF receptor induced apoptosis only when FGFR2 siRNA was included (Figure 3B, left bottom graph). In MLS 1765 cells knock-down of FGFRs induced apoptosis as well, but to a lesser extent than in MLS 402. Silencing of FGFR2 alone did not cause apoptosis, but apoptosis induction was detected upon knock-down of either FGFR1 or FGFR3 (Figure 3B, right upper graph). In line with this all combinations of different siRNAs including those against FGFR1 and/or FGFR3 induced apoptosis in MLS 1765 cells (Figure 3B, right bottom graph).

In MLS 402 cells the combined silencing of the four FGF receptors caused attenuation of ERK mediated downstream signaling, shown by the reduced phosphorylation of ERK1/2 (Figure 3C). In MLS 1765 cells even single receptor silencing of either FGFR1 or FGFR3 lead to a reduction of ERK1/2 phosphorylation (Figure 3C). This result is in concordance with apoptosis induction in MLS 1765 cells after silencing of FGFR1 or FGFR3.

### FGFR inhibition with small molecules

**Figure 4:**
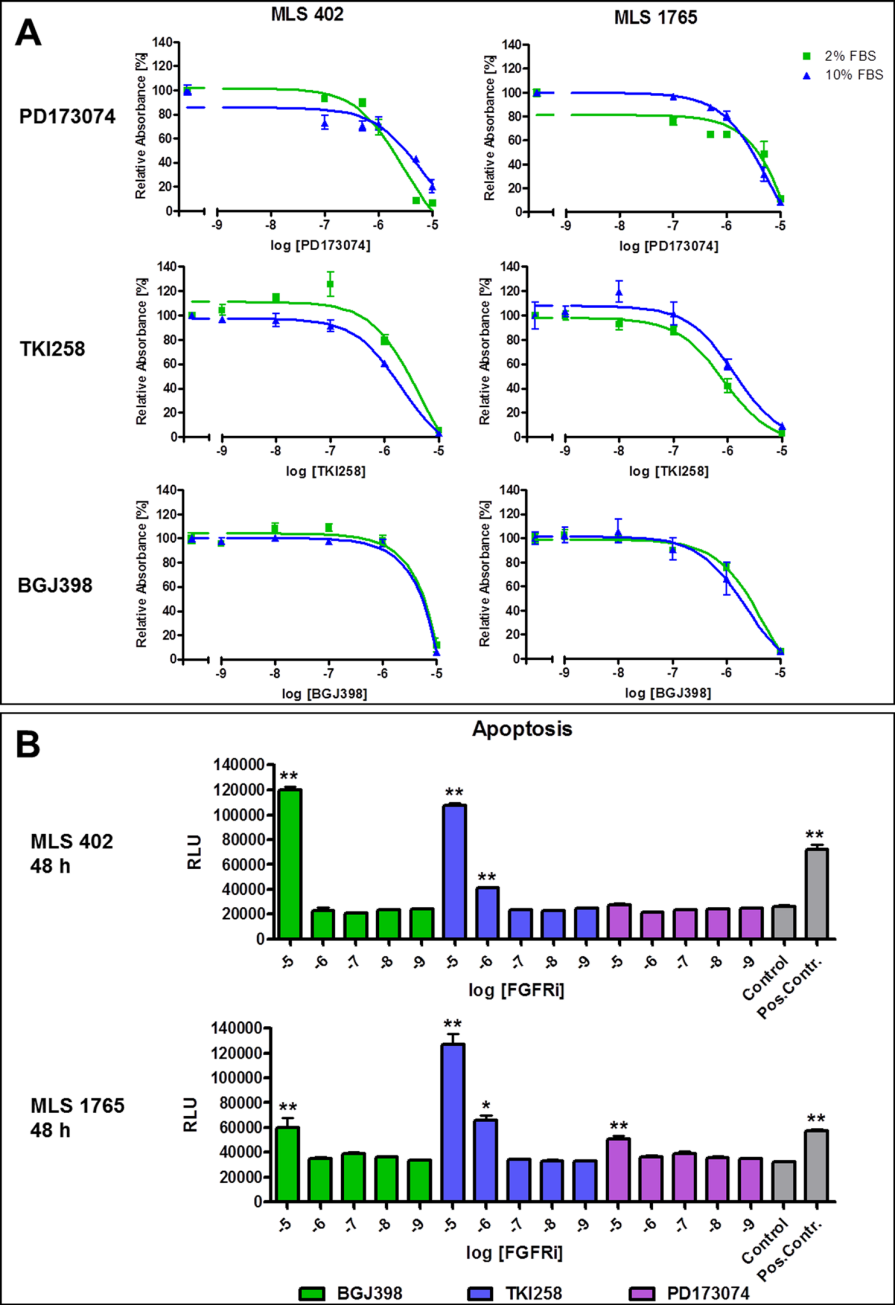
Effects of FGFR inhibitors on cell viability and apoptosis induction **A.** Effects of FGFR inhibition with PD173074, TKI258 and BGJ398 on the viability of MLS cell lines. Cells were treated with different inhibitor concentrations under full serum (10% FBS) or serum reduced (2% FBS) conditions and analyzed using MTT assay after 48 h. Data of quintuplicates are represented as mean +/− SEM. **B.** Apoptosis induction in MLS cell lines 48 h after treatment with FGFR inhibitors. As negative control cells were treated with 0.1% DMSO. Camptothecin treated cells served as positive control for apoptosis induction (Pos.Contr.). FGFRi: FGFR inhibitors. Data of quintuplicates are represented as mean +/− SEM. *: *p* ≤ 0.05; **: *p* ≤ 0.01; ***: *p* ≤ 0.001.

As silencing of FGF receptors with specific siRNAs induced apoptosis in the myxoid liposarcoma cell lines MLS 402 and MLS 1765, also the functional effects of small molecules directed against FGFRs were examined. Cells were treated with the *in vitro* compound PD173074 (purchased from Sigma Aldrich) as well as with the two clinically applicable FGFR inhibitors dovitinib (TKI258) and BGJ398 (both from Novartis). As shown in Figure 4A, viability of both cell lines was reduced through FGFR inhibition by all three compounds in a concentration dependent manner. IC50 values were in the micromolar range and inhibitory effects could be seen under serum-reduced as well as under full-serum conditions. The detected effects upon FGFR inhibition by TKI258 and BGJ398 on the viability of the MLS cell lines were compared to those in a known FGFR inhibitor sensitive cell line. For that purpose the *FGFR2* amplified gastric cancer cell line Kato-III was used. This cell line was markedly more sensitive to the highly FGFR specific compound BGJ398, whereas the sensitivity towards the more broad-spectrum tyrosine kinase inhibitor TKI258 was similar in MLS and Kato-III cells (Supplemental Figure S4A).

Effects of FGFR inhibitors on the viability of myxoid liposarcoma cell lines were further differentiated using the ApoTox-Glo™ Triplex Assay (Promega). Inhibitor concentrations reducing the viability of the cells (1 – 10 μM) were proven to be not cell toxic, but specifically inducing apoptosis (Figure 4B). BGJ398 and TKI258 already induced apoptosis after 48 hours of treatment in both cell lines, whereas apoptosis induction upon treatment with PD173074 in MLS 402 was detected only after 72 hours (Supplemental Figure S4B).

**Figure 5:**
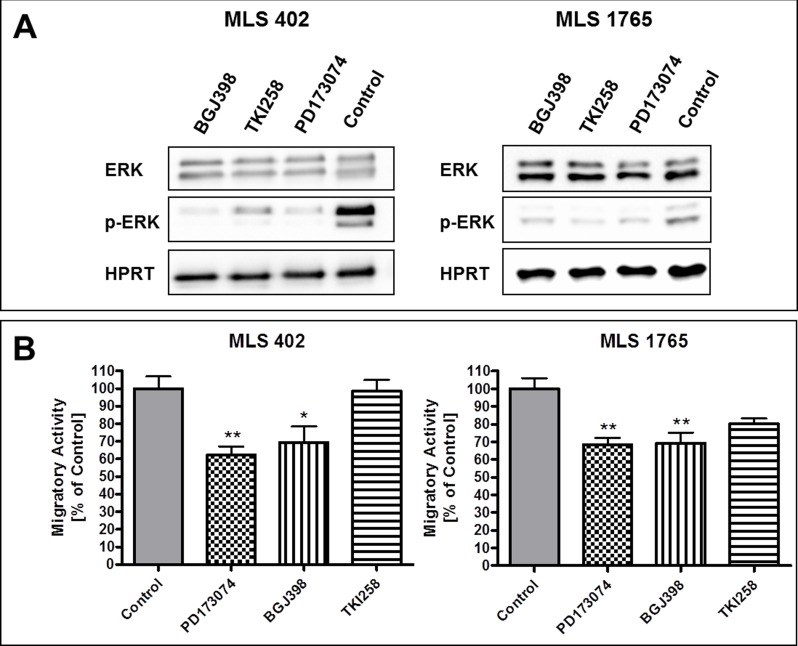
Effects of FGFR inhibitors on downstream signaling and cell migration **A.** Effects of FGFR inhibitors [1 μM] on downstream signaling through phosphorylation of ERK1/2. Protein lysates were collected 5 h after treatment with the respective inhibitor. Lysates of cells treated with 0.1% DMSO served as controls. **B.** Effects of FGFR inhibitors [0.1 μM each] on the migratory activity of MLS cell lines. Control cells were treated with 0.1% DMSO. Data of triplicates are represented as mean +/− SEM. *: *p* ≤ 0.05; **: *p* ≤ 0.01; ***: *p* ≤ 0.001.

FGFR inhibition in myxoid liposarcoma cell lines reduced kinase activity of the receptors, as detected by reduced receptor phosphorylation (Supplemental Figure S5A). Furthermore, the downstream signaling was affected, as shown exemplarily for ERK1/2 (Figure 5A). All three FGFR inhibitors reduced the phosphorylation of ERK1/2, being one of the main downstream targets of FGFRs.

Additionally, the effects of FGFR inhibition on the migratory activity of MLS 402 and MLS 1765 cells were examined by scratch assay. The FGFR inhibitors PD173074, BGJ398 and TKI258 were applied in a final concentration of 0.1 μM, a concentration which does not affect cell viability in the regarded timeframe of 24 hours. Supplemental Figure S5B exemplarily shows the effects of BGJ398 on the migration of MLS 1765 cells. Scratch closure was examined in FGFR inhibitor treated and DMSO treated control cells and the migratory activity was calculated as relative scratch closure compared to control. All three FGFR inhibitors reduced the migratory activity of both myxoid liposarcoma cell lines (Figure 5B). However, PD173074 and BGJ398 showed much stronger effects than TKI258, which had only little impact on the migration of MLS 402 cells. In MLS 1765 the effect of TKI258 on cell migration was indeed more obvious but still not significant.

### Combination of FGFR inhibitors with trabectedin

**Figure 6:**
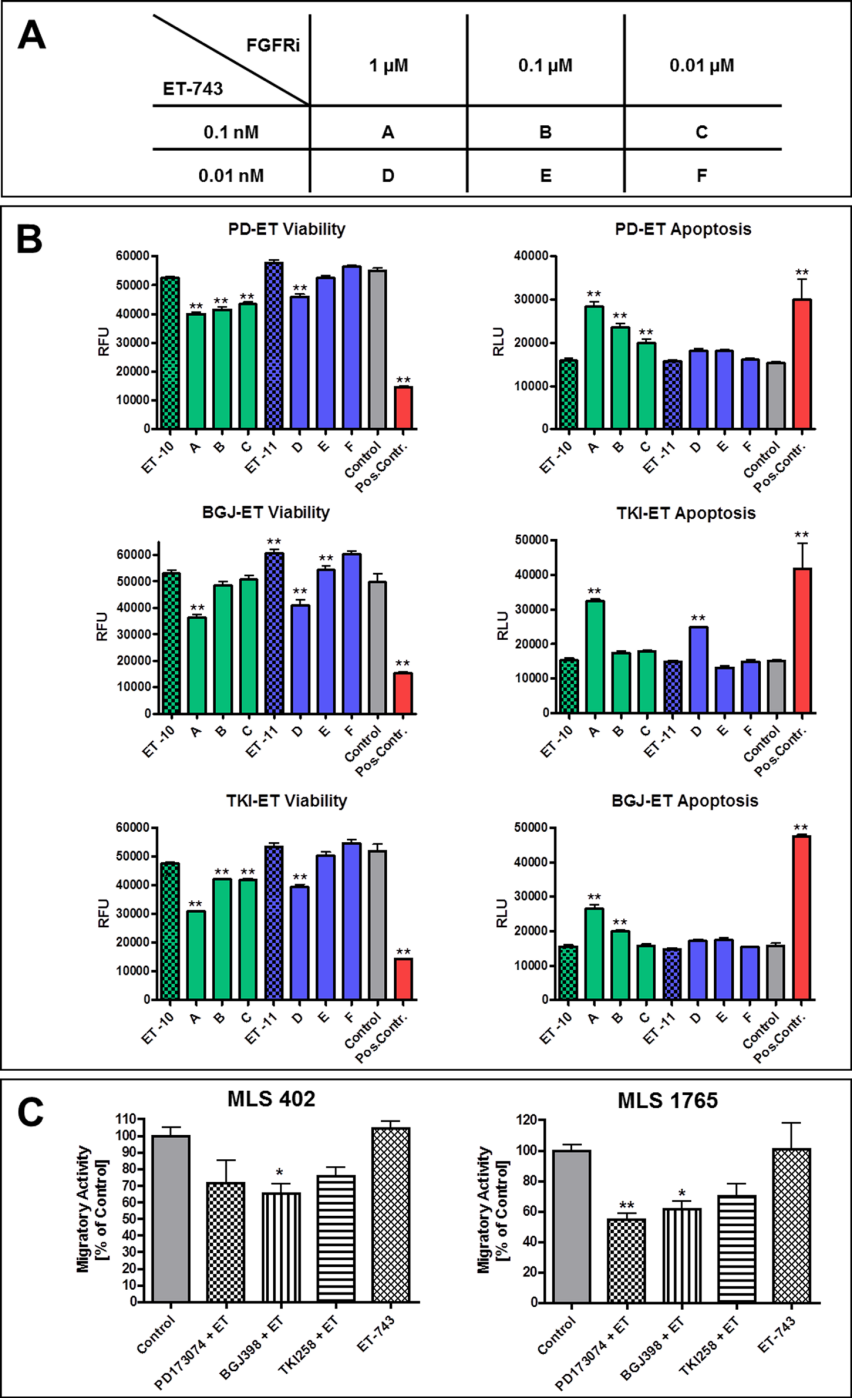
Combined treatment with FGFR inhibitors and trabectedin **A.** Combination scheme of FGFR inhibitors and trabectedin. **B.** Effects of combined treatment with FGFR inhibitors together with trabectedin on MLS 1765 cells in comparison to trabectedin alone. Cells were treated with different combinations of compound concentrations (as shown in A) and effects on cell viability as well as induction of apoptosis were determined after 48 h. As negative control cells were treated with 0.2% DMSO. Digitonin and camptothecin treated cells served as positive controls for reduction of viability and induction of apoptosis, respectively. Data of quintuplicates are represented as mean +/− SEM. *: *p* ≤ 0.05; **: *p* ≤ 0.01; ***: *p* ≤ 0.001. **C.** Migratory activity of MLS 402 and MLS 1765 cells under treatment with trabectedin [0.1 nM] alone or combined with FGFR inhibitors [0.1 μM each]. Control cells were treated with 0.2% DMSO. Data of triplicates are represented as mean +/− SEM. *: *p* ≤ 0.05; **: *p* ≤ 0.01; ***: *p* ≤ 0.001. ET-743 / ET: trabectedin, FGFRi: FGFR inhibitors, PD: PD173074, BGJ: BGJ398, TKI: dovitinib (TKI258).

Additionally to single agent treatment, FGFR inhibitors were applied in combination with trabectedin (ET-743). The corresponding combination scheme is given in Figure 6A. As shown in Figure 6B for MLS 1765, the supplementary administration of FGFR inhibitors enhanced the effects of trabectedin in comparison to single agent treatment. Cell viability was reduced and apoptosis was induced by all three inhibitors in combination with trabectedin in a concentration below its single agent efficacy. The same holds true for the migratory activity of myxoid liposarcoma cell lines. In MLS 402 as well as in MLS 1765 cells migration was delayed by the additional administration of FGFR inhibitors when compared to cells treated with trabectedin alone (Figure 6C). As seen in the single agent migration assay, PD173074 and BGJ398 displayed a greater impact on the migration of myxoid liposarcoma cells than TKI258.

## DISCUSSION

The treatment of soft tissue sarcomas in general and of myxoid liposarcomas in particular is so far restricted to unspecific chemotherapy and preferably total surgical resection. The implementation of new specific therapies requires the identification of suitable targetable molecular structures. An approach to reveal such target structures is comparative gene expression profiling as applied in this study by whole-genome microarray analyses on seven myxoid liposarcoma samples and an RNA pool of eight fat tissue samples. That way differential expression of multiple genes was identified. A principal component analysis demonstrated a joint clustering of the tumor samples apart from the control sample. We detected nearly 8,000 genes with a significant fold change of at least ±2. Compared to other gene expression profiling studies in sarcomas the number of differentially expressed genes identified in this study is rather high, but previous studies mainly compared different sarcoma entities to each other and did not include whole-genome expression analyses [11, 12, 14]. Furthermore, the high number of differentially expressed genes demonstrates the strong homogeneity in our tumor cohort. This feature of myxoid liposarcomas became already apparent in previous gene expression and methylation profiling studies that observed tight clusters for myxoid liposarcomas clearly distinguishable from other entities or corresponding normal tissue [12-14].

In order to confirm the reliability of microarray data by qPCR validation, it is important to determine suitable references because a universal reference for all kinds of tissues and experimental conditions does not exist [24-27]. Our data revealed that widely used reference genes, such as *GAPDH*, are not self-evidently appropriate for formalin fixed tissue with intense degradation of RNA. We could identify *B2M* and *IPO8* as suitable reference genes for myxoid liposarcoma and fat control samples in cryo-conserved as well as in formalin-fixed tissue. The herein presented whole-genome expression analyses could be reproduced very well by means of qPCR even by using FFPE tissue and served therefore as method to identify differentially expressed candidate genes.

Receptor tyrosine kinases belong to the most promising target structures as their constitutive activation has been shown in various tumor entities and they can successfully be targeted [19, 28, 29]. A constitutively active receptor tyrosine kinase may be due to DNA mutations or gene amplification but as well overexpression can lead to permanent receptor signaling [30, 31]. The present study has revealed the receptor tyrosine kinase *fibroblast growth factor receptor 2* (*FGFR2*) as a gene of interest being highly overexpressed in myxoid liposarcoma.

FGF receptors were already reported to play a role in various cancers and their inhibition is effectively applied in targeted therapies with specific small molecule inhibitors [32-38]. Even in sarcomas a role of FGF receptors was described. Girnita et al. showed the importance of the bFGF pathway for the maintenance of a malignant phenotype of Ewing's sarcoma cells [39]. The functional role and the effective inhibition of FGF receptors in synovial sarcoma were shown by Ishibe et al. [40]. A recent study in canine and human sarcomas detected co-expression of various FGFRs and demonstrated growth inhibitory effects by FGFR inhibition *in vitro* [41]. An expression study in different sarcomas by Baird et al. revealed an association of *FGFR2*, *FGFR4* and *FGF18* gene expression with liposarcomas [11]. This association became also apparent in the present study where we could detect overexpression not only of *FGFR2* but also of *FGFR4* as well as of the ligands *FGF5*, *FGF11* and *FGF18*. The parallel overexpression of several FGF/FGFR family members reinforces a potential role of FGFR signaling in the pathogenesis of myxoid liposarcoma. Furthermore, FGFR signaling is described as being essential in preadipocyte differentiation providing another possible connection to the pathogenesis of liposarcomas that are characterized by the presence of premature adipocytes [42].

*In vitro* experiments in myxoid liposarcoma cell lines confirmed a functional role of FGFR signaling. The administration of specific siRNAs directed against FGFRs induced apoptosis in MLS 402 and MLS 1765 cells whereby the two cell lines reacted differently to selective FGFR silencing. While cell survival in MLS 402 cells seems to be dependent on FGFR2, in MLS 1765 rather FGFR1 and FGFR3 are important. Thus, in myxoid liposarcoma cells there is obviously not a single FGF receptor acting as a strong tumor driver. Accordingly, small molecules inhibiting the whole group of FGF receptors reduced cell viability, induced apoptosis and delayed migration in both myxoid liposarcoma cell lines.

In the examined myxoid liposarcoma cell lines the impact of FGFR signaling on survival and migration seemed to outweigh that on proliferation. FGFR inhibitor concentrations needed to achieve the observed effects differed in at least one decimal power. Furthermore, the FGFR specific inhibitors PD173074 and BGJ398 had a much greater impact on cell migration than the rather broad-spectrum tyrosine kinase inhibitor TKI258 also sustaining the hypothesis that in myxoid liposarcoma the role of FGFR signaling is especially prominent for cell migration. The herein used FGFR inhibitors TKI258 and BGJ398 are already applied in clinical trials of other tumor entities, such as *FGFR3* mutated bladder cancer or squamous cell lung cancer with *FGFR1* amplification (e.g. NCT01004224 or NCT01831726). We now demonstrated FGFR overexpression in myxoid liposarcoma and the effective application of FGFR inhibition *in vitro* indicating a novel targeted therapy approach. This approach might involve the combination of FGFR inhibitors with chemotherapeutic agents like trabectedin. In this study we showed that the additional administration of FGFR inhibitors can improve the impact on myxoid liposarcoma cells in comparison to trabectedin alone.

In order to examine whether FGFR inhibitors alone or in combination with chemotherapy are efficient as a potent therapy in myxoid liposarcoma, further *in vivo* studies are needed. In an *in vivo* setting also the role of FGFR signaling in angiogenesis will surely become relevant. Several FGFs are described to promote neoangiogenesis, among them FGF2 but also FGF5 and FGF18, for which an overexpression in myxoid liposarcomas was detected in this study [43-45]. Given the fact that myxoid liposarcomas are characterized by a typical branching capillary network, an inhibitor blocking also angiogenic pathways might be particularly effective in this entity.

In conclusion, our study revealed overexpression of *FGFR2* as well as a functional role of FGFR signaling in myxoid liposarcoma and provides basis for the use of FGFR inhibitors as a novel targeted treatment approach for these tumors. Further *in vivo* studies are warranted to affirm the efficacy of FGFR inhibition either alone or in combination with chemotherapy.

## MATERIALS AND METHODS

### Human tissue samples

A cohort of 16 human primary myxoid liposarcomas and 15 fat tissue samples was assorted (Table 1). Tumor samples were provided by the Biobank of the Center for Integrated Oncology (CIO) Cologne Bonn and by the Institute of Pathology in Heidelberg. Fat tissue samples were obtained during intra-operative diagnostics in the scope of the CIO Cologne Bonn. Cryo-conserved material of eight fat tissue samples was additionally post-fixed in formalin and paraffin-embedded (FFPE) to obtain suitable controls for FFPE tumor samples. The study was approved by the ethics committees of the medical schools of the universities Bonn and Cologne. All cases were histologically characterized by an experienced pathologist (HUS, GM, EW or RB) and *DDIT3*-translocation was proven with fluorescence in-situ hybridization (FISH) and reverse transcription (RT)-PCR. FISH probes for *DDIT3*, *FUS* and *EWS1R* were from Abbott Molecular (Abbott Park IL, U.S.A.). Primer sequences for RT-PCR reactions are given in Supplemental Table S1.

### RNA extraction, quality control and cDNA synthesis

Total RNA extraction was performed with miRNeasy mini or miRNeasy FFPE Kit (both Qiagen, Hilden, Germany). RNA was quantified using Nanodrop 1000 spectrophotometer (Thermo Scientific, Waltham MA, U.S.A.). Assessment of RNA degradation was performed via Agilent 2100 Bioanalyzer and RNA 6000 Nano Assay (Agilent Technologies, Santa Clara CA, U.S.A.). cDNA was synthesized using random hexamer primers and Omniscript Reverse Transcription Kit (Qiagen). Two independent cDNA syntheses were pooled for each sample. In order to detect FGFR knock-down after transfection of specific siRNAs the High-Capacity RNA-to-cDNA™ Kit (Life Technologies, Carlsbad CA, U.S.A.) was used for cDNA synthesis.

### Microarray analyses

RNA from seven tumor samples and an RNA pool of eight fat tissue samples with a minimal RIN value of 7.0 were selected for whole-genome microarray analyses with Agilent Whole Human Genome Oligo Microarrays 4×44K. Microarray analyses were carried out by the genomic service of Miltenyi Biotec (Bergisch Gladbach, Germany). Raw data of microarray hybridizations have been deposited in NCBI's Gene Expression Omnibus [46] and are accessible through GEO Series accession number GSE62747 (http://www.ncbi.nlm.nih.gov/geo/query/acc.cgi?acc=GSE62747).

### Quantitative real-time PCR (qPCR)

qPCR reactions were performed using Applied Biosystems 7900HT Fast Real-Time PCR System (Life Technologies) or LightCycler^®^ 480 II system (Roche Diagnostics, Rotkreuz, Switzerland). Expression stability of 16 potential reference genes was analyzed with TaqMan^®^ Array Human Endogenous Control Panels (Life Technologies). Obtained raw Cq (cycle of quantification) values were converted into relative quantities and analyzed using GeNorm [47] and NormFinder (MDL, Aarhus, Denmark). Expression of candidate genes was analyzed using Power SYBR^®^ Green PCR Kit (Life Technologies). Corresponding primer pairs are given in Supplemental Table S2. Gene expression data were analyzed using the Relative Expression Software Tool (REST) [48] and graphically visualized using GraphPad Prism (GraphPad Software, La Jolla CA, U.S.A.). In order to detect FGFR knock-down in MLS cell lines after siRNA transfection TaqMan^®^ Gene Expression Assays for *FGFR1, 2, 3,* and *4* as well as the reference genes *IPO8* and *B2M* were used along with the TaqMan^®^ Universal Master Mix II (all from Life Technologies).

### FGFR2 expression in tissue samples

Gene expression of *FGFR2* in primary tumor samples was detected by qPCR with Power SYBR® Green PCR Kit (Life Technologies) and specific primers (for 5′-GTGAAACTTGGTACTTCATGGTGA, rev 5′-GAGATGGCATTCTTGTTGTTACTG). Expression data were normalized using *IPO8* and *B2M* as reference genes and relative gene expression was calculated with REST. Immunohistochemistry for FGFR2 was performed using rabbit anti-FGFR2 antibody from Zytomed Systems (Berlin, Germany). FGFR2 protein expression in primary tumor and fat tissue samples was analyzed with western blot using rabbit anti-FGFR2 antibody from Sigma Aldrich (St. Louis MO, U.S.A.) in a dilution of 1:500. For detection of reference protein expression rabbit anti-HPRT antibody from abcam (Cambridge, GB) in a dilution of 1:1000 was used.

### Cell culture and FGFR inhibition *in vitro*

Myxoid liposarcoma cell lines MLS 402 and MLS 1765 were provided by Prof. Pierre Åman, University of Gothenburg, Sweden and cultured in RPMI 1640 medium supplemented with 2 mM L-glutamine and 2% or 10% FBS under humidified conditions with 5% CO_2_. Cell lines' entity was verified by proof of the specific *FUS-DDIT3* translocation. Furthermore, uniqueness of the cell lines was confirmed by STR profiling performed at DSMZ, Braunschweig, Germany. Gastric cancer cell line Kato-III was purchased from Cell Lines Service (Eppelheim, Germany) and cultured in F12 medium supplemented with 2 mM L-glutamine and 10% FBS under humidified conditions with 5% CO_2_. *FGFR2* amplification in Kato-III cells was proven with FISH analysis using Zytolight SPEC FGFR2/CEN 10 Dual Color Probe from ZytoVision (Bremerhaven, Germany).

FGFR inhibitors PD173074 (purchased from Sigma Aldrich), BGJ398 and TKI258 (both provided by Novartis, Basel, Switzerland) as well as trabectedin (provided by PharmaMar, Madrid, Spain) were solved and prediluted in DMSO. Final DMSO concentration during assays was 0.1% for single agent or 0.2% for combined treatment. For FGFR silencing cells were transiently transfected with 100 nM siRNA using Lipofectamine™ RNAiMax (Life Technologies) and Stealth RNAi™ siRNAs against FGFR1, 2, 3 and 4 (set of 3 siRNAs each) as well as Stealth RNAi™ Negative Control Duplexes (all from Life Technologies). AllStars Hs Cell Death siRNA (Qiagen) served as transfection control.

### Detection of cell viability, toxicity and apoptosis

Cell viability was measured in quintuplicate using MTT assay. 10 μl MTT staining solution (5 mg/ml in PBS, sterile filtered) were added to each well of a 96well plate and reaction was stopped after 5 h with 100 μl MTT solvent (10% SDS in 0.01 M HCl). Formed crystals were lyzed overnight at 37°C and absorbance was detected at 550 nm deducting background at 690 nm. To further differentiate observed effects cells were analyzed using ApoTox-Glo™ Triplex Assay (Promega, Madison WI, U.S.A.).

### Western blot

Total protein lysates were extracted from untreated cell lines as well as 48 h after siRNA transfection or 2 or 5 h after inhibitor treatment. For western blot analysis equal amounts of total protein were loaded on NuPAGE^®^ Bis-Tris Gels (Life Technologies). Electrophoretically separated proteins were transferred to nitrocellulose membrane and unspecific binding was blocked with 5% milk/PBS-Tween. Membranes were incubated with primary antibodies overnight at 4°C (FGFR2 1:500; ACTB 1:5000, AC-15, both Sigma Aldrich; FGFR1 1:500; FGFR4 1:500, AM11076PU-N, both from Acris Antibodies, San Diego CA, U.S.A.; FGFR3 1:500, D2G7E; phospho-FGFR 1:500, 55H2; ERK1/2 1:1000; phospho-ERK1/2 1:1000, 20G11, all from CellSignaling Technology, Danvers MA, U.S.A.; HPRT 1:1000, abcam). After incubation with HRP-conjugated secondary antibody SuperSignal West Pico or Femto Chemiluminescent Substrate (Thermo Scientific) was used for detection.

### Scratch assay

Cells were grown to 100% confluence in medium containing 10% FBS in 24 well culture plates. In each well a scratch was set crosswise with a 100 μl pipet tip, debris was washed away using PBS. Cells were incubated for 24 h with medium containing 2% FBS and the respective treatment. Pictures of each well with centered scratch were taken before and after treatment period and uncovered areas were determined using AxioVision 4.8 software (Carl Zeiss, Oberkochen, Germany). Scratch closure was calculated as the proportion of previously uncovered area closed by cell migration after the considered period. Migratory activity was defined as relative scratch closure compared to control.

## SUPPLEMENTARY FIGURES AND TABLES


