# Supplementary material for: FGFR2 is overexpressed in myxoid liposarcoma and inhibition of FGFR signaling impairs tumor growth in vitro
Source: Oncotarget. 2015 May 8;6(24):20215–30. doi: 10.18632/oncotarget.4046 (PMC4652999; doi:10.18632/oncotarget.4046)
Supplement: Supplementary file 1 [file oncotarget-06-20215-s001.pdf]

FGFR2 is overexpressed in myxoid liposarcoma and inhibition of FGFR signaling impairs tumor growth *in vitro*

Supplementary Material

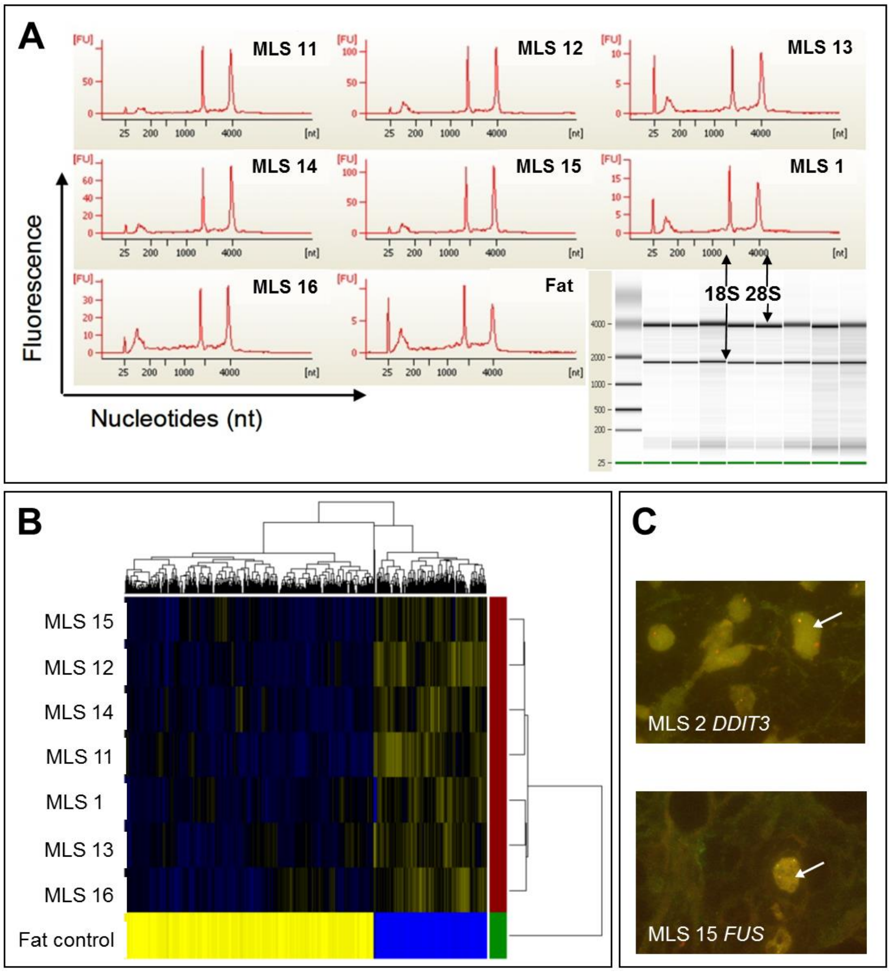

Figure S1: Whole-genome microarrays: sample quality and heat map

A Excellent RNA quality of samples used for microarray analyses, apparent by the two peaks or bands representing the 18S and 28S rRNA.

B Heat map of the gene expression profiles showing the differential expression between fat control pool and tumor samples as detected with whole-genome microarray analyses. Blue color indicates low gene expression whereas highly expressed genes are displayed in yellow.

C FISH analyses in primary myxoid liposarcomas confirm differential diagnosis; arrows indicate cells with characteristic break-apart patterns showing the translocation event.

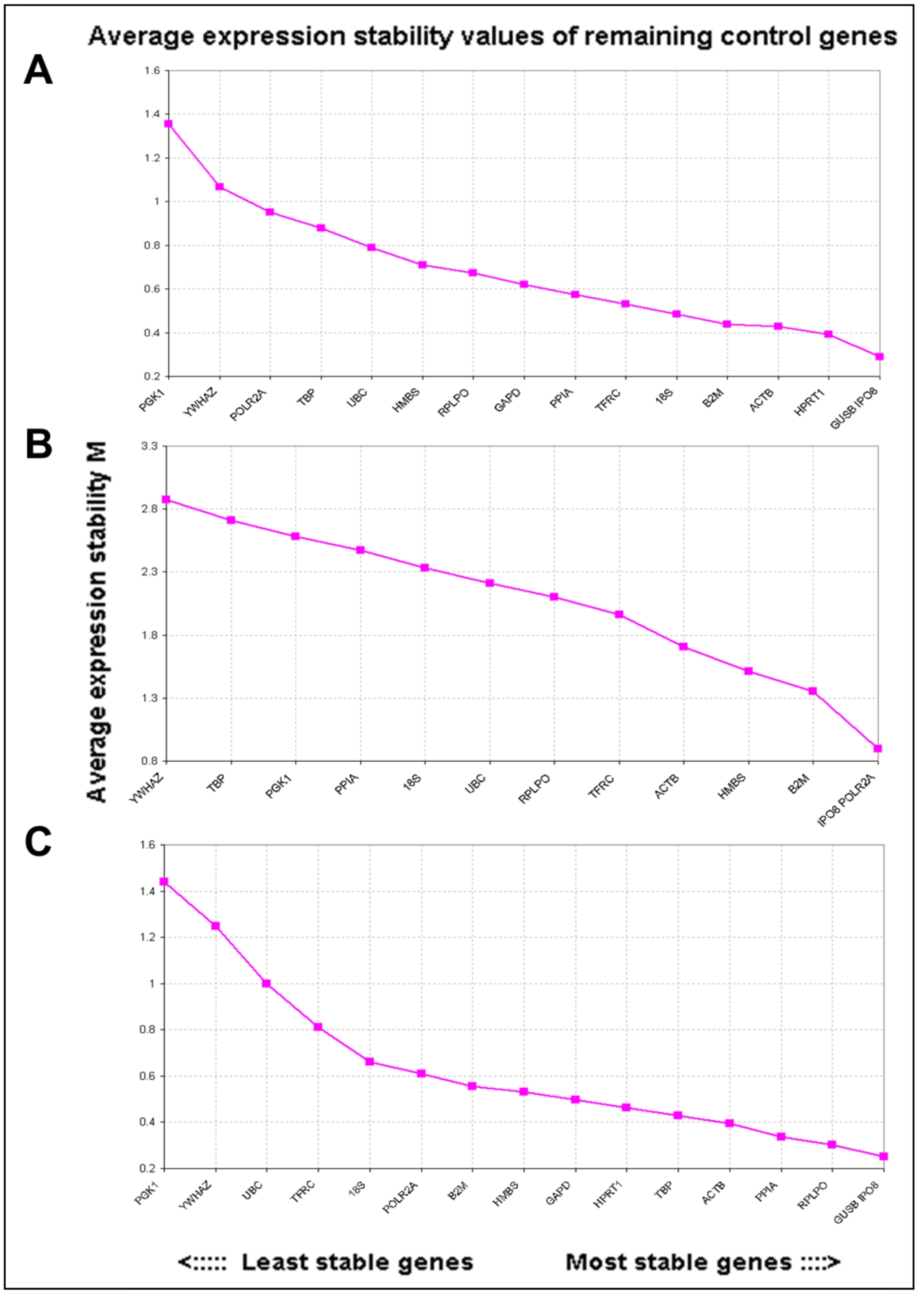

Figure S2: Expression stability of reference genes

Expression stability of candidate reference genes as generated with the GeNorm software tool.

A Expression stability values in cryo-conserved tumor samples.

B Expression stability values in FFPE tumor samples. As expression of *GAPDH*, *GUSB* and *HPRT1* was not detected in all formalin-fixed tumor samples, they were excluded from subsequent analyses.

C Expression stability values in cryo-conserved fat tissue samples.

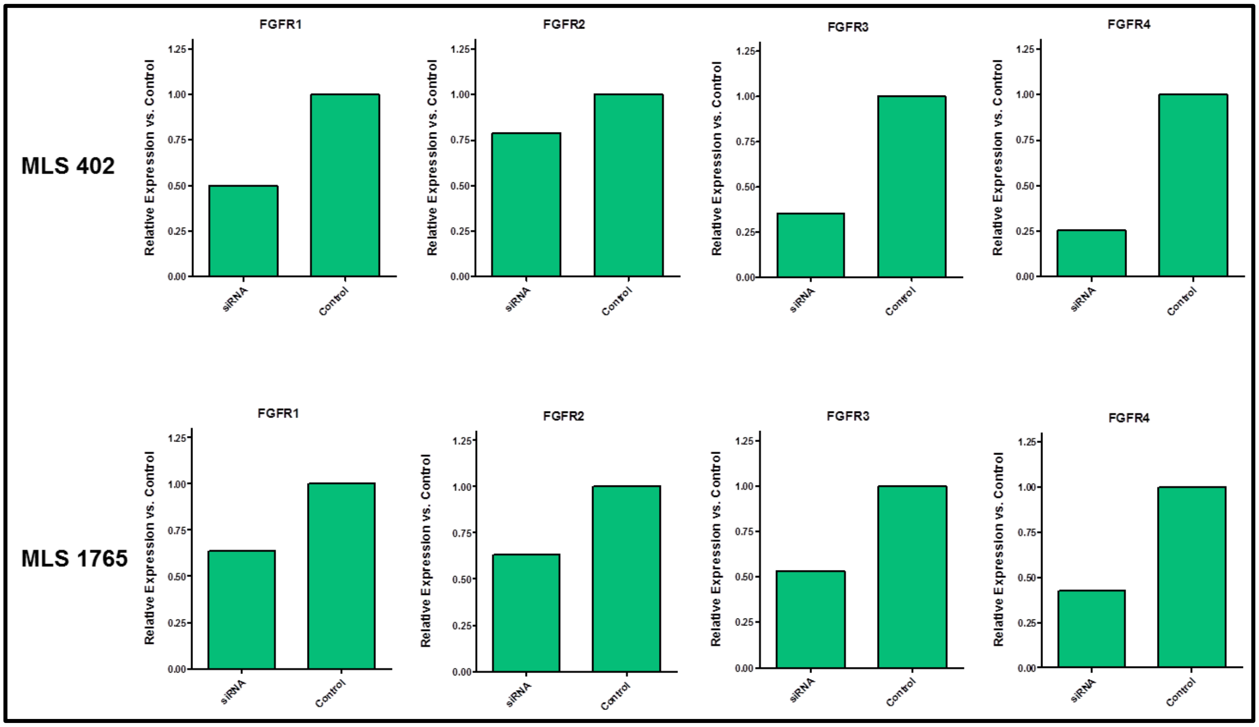

Figure S3: FGFR Knock-down in MLS cell lines

qPCR for *FGFR1*, *FGFR2*, *FGFR3* and *FGFR4* in MLS cell lines 48 h after transfection with specific siRNAs. As negative control cells were transfected with Stealth RNAi™ Negative Control Duplexes with the corresponding GC content.

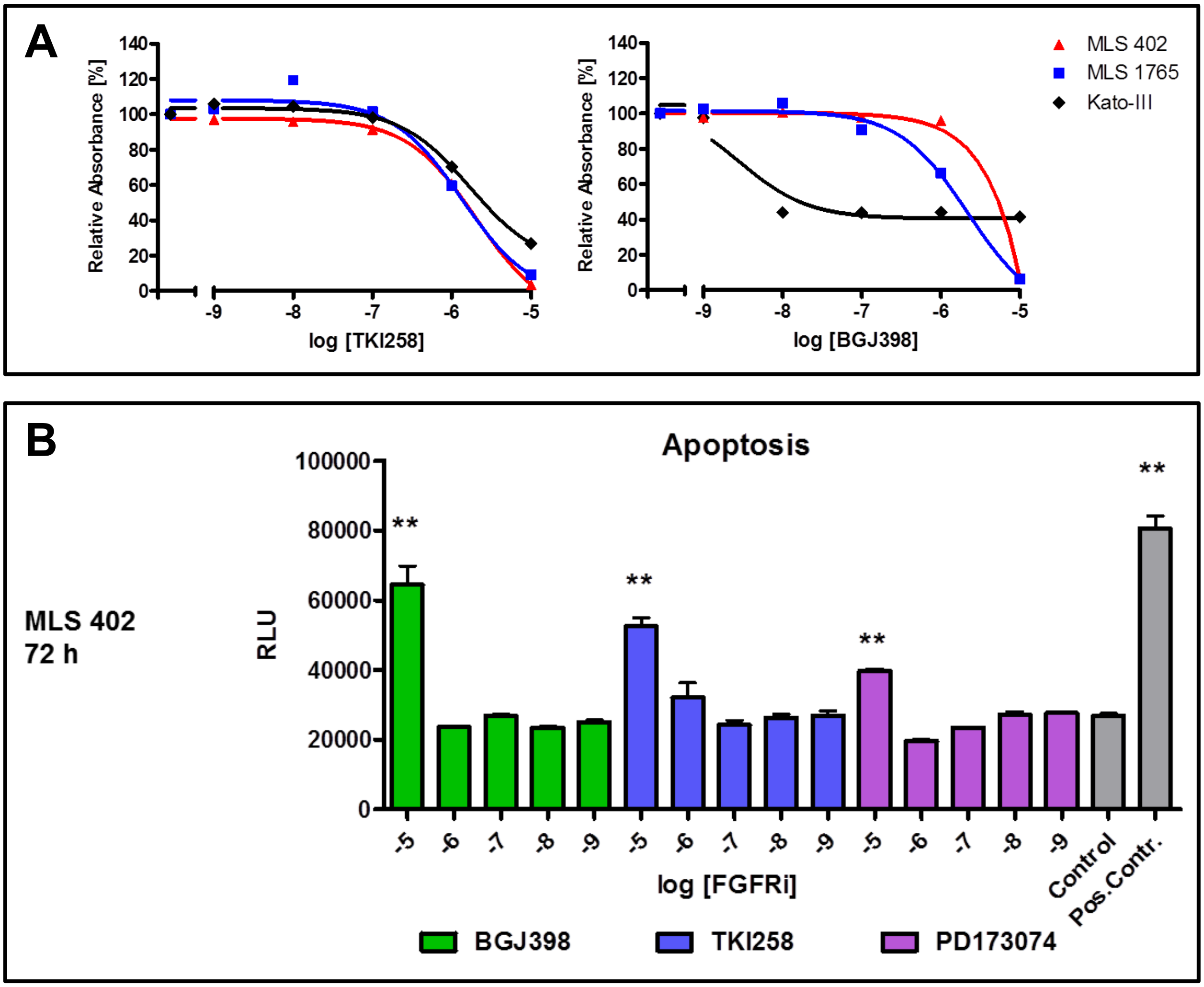

Figure S4: Effects of FGFR inhibitors on myxoid liposarcoma cells

A Effects of FGFR inhibition with TKI258 and BGJ398 on the viability of MLS cell lines in comparison to Kato-III cells. Cells were treated with different inhibitor concentrations and analyzed using MTT assay after 48 h.

B Apoptosis induction in MLS 402 cells 72 h after treatment with FGFR inhibitors (FGFRi). As negative control cells were treated with 0.1% DMSO. Camptothecin treated cells served as positive control for apoptosis induction. Data of quintuplicates are represented as mean +/- SEM. *: $p \leq 0.05$; **: $p \leq 0.01$; ***: $p \leq 0.001$.

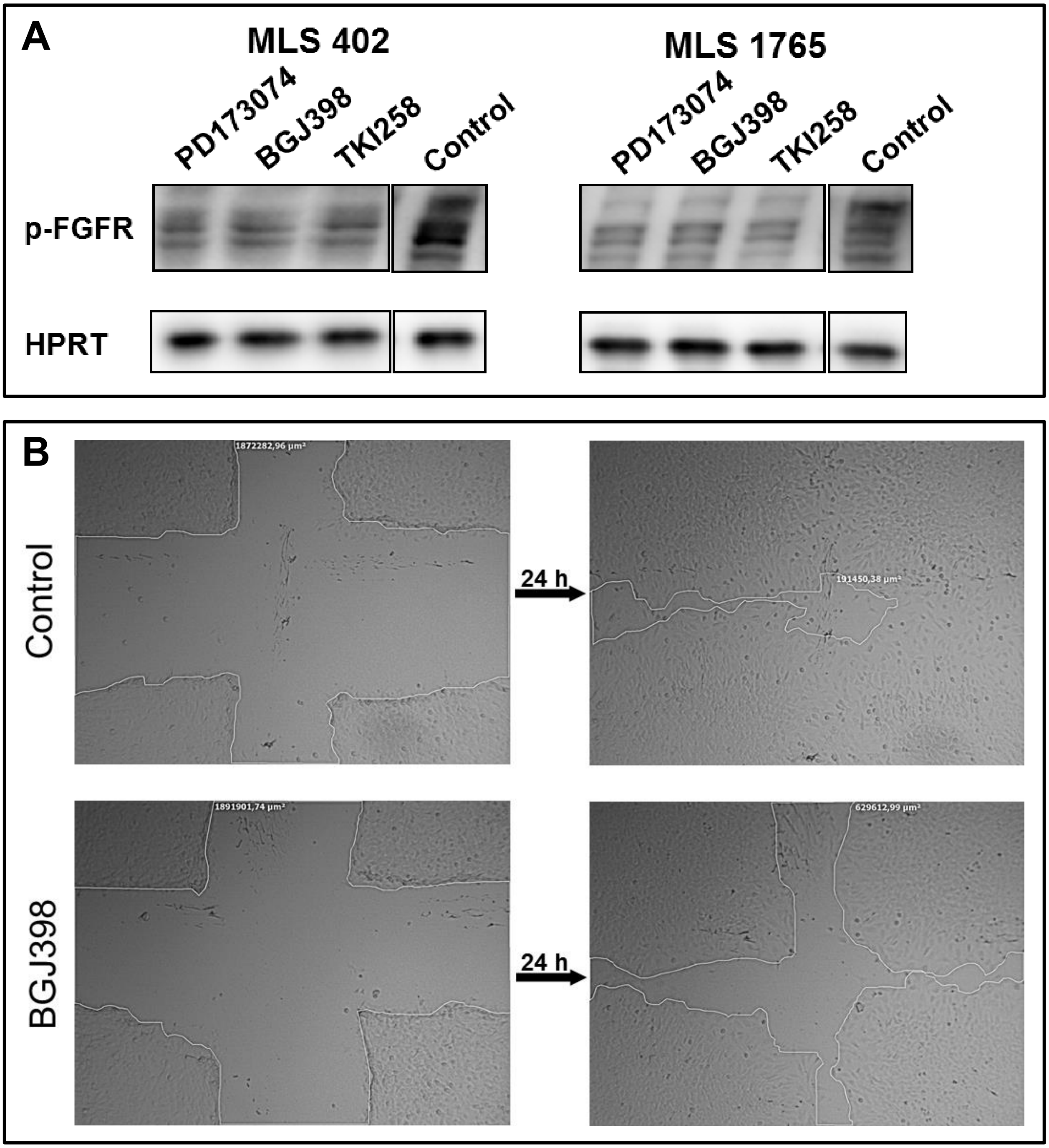

Figure S5: Attenuation of FGFR signaling and delay of cell migration

A Effects of FGFR inhibitors [1 µM] on FGFR kinase activity. Protein lysates were collected 2 or 5 h after treatment with the respective inhibitor. Lysates of cells treated with 0.1% DMSO served as controls.

B Effects of FGFR inhibition by BGJ398 [0.1 µM] on the migration of MLS 1765 cells. Control cells were treated with 0.1% DMSO.

Table S1: Detection of *FUS-DDIT3* fusion transcripts

In MLS cell lines and cryo-conserved tumor samples the exact type of fusion transcript was detected by RT-PCR and subsequent sequencing.

A Primer for RT-PCR

**Fusion Transcript**	**Primer Sequence [5'-3']**	
FUS-DDIT3 I	Forward	GTGGTTACAACCGCAGCAGTG
	Reverse	AAGGAGAAAGGCAATGACTCAG
FUS-DDIT3 II	Forward	CCTAGCTATGGTGGACAGCAG
	Reverse	AAGGAGAAAGGCAATGACTCAG
FUS-DDIT3 III	Forward	GTGGCTATGAACCCAGAGGTC
	Reverse	AAGGAGAAAGGCAATGACTCAG

B Types of fusion transcripts

**Sample**		**Type of Fusion Transcript**
MLS 402	Cell line	Type I (7-2)
MLS 1765	Cell line	Type VIII (14-2) *
MLS 1	Primary tumour	Variant of type I (lack of last 24 codons of *FUS* exon 7)
MLS 11	Primary tumour	Type III (8-2)
MLS 12	Primary tumour	Type I (7-2) and type III (8-2)
MLS 13	Primary tumour	Type III (8-2)
MLS 14	Primary tumour	Type I (7-2)
MLS 15	Primary tumour	Type III (8-2)
MLS 16	Primary tumour	Type III (8-2)

* Information from Prof. Pierre Åman, University of Gothenburg, Sweden – detection not possible with the primer design used in this study. MLS: myxoid liposarcoma; numbers in brackets describe fused *FUS* and *DDIT3* exons.

Table S2: Primer for validation of candidate genes

Gene Symbol	Primer Sequence [5'-3']	
IPO8	Forward<br>Reverse	TACTATGTGGAGATGCAGGAGAAG<br>AACAAGTTGAACGAAGAGTGGAAT
B2M	Forward<br>Reverse	CAGCAAGGACTGGTCTTTCTATCT<br>CCATGATGCTGCTTACATGTCT
CIDEA	Forward<br>Reverse	ATGATCTTGGAAAAAGGACAGAAG<br>CTGTACAAGTCGAAGGTGACTCTC
IL6	Forward<br>Reverse	CTGTGCAGATGAGTACAAAAGTCC<br>ATGAGATGAGTTGTCATGTCCTG
MAGEA4	Forward<br>Reverse	GGGTTAGAGAGAAGCGAGCTG<br>TCTCCTTGGTGCTCCTCTGT
PRAME	Forward<br>Reverse	AGTTCACCTCTCAGTTCCTCAGTC<br>GGCAGTTAGTTATTGAGAGGGTTT
SOCS3	Forward<br>Reverse	CAAGGACGGAGACTTCGATTC<br>AACTTGCTGTGGGTGACCAT
SOX11	Forward<br>Reverse	ACCTGAGCTTGAATTTCTCTCAAA<br>AACGAATCCAAATCCTTATCCAC
SSX4B	Forward<br>Reverse	CCAAGTACCTTGGAGAAGATTAACA<br>GCTGATCTCTTCATAAACCACCAG
TOP2A	Forward<br>Reverse	GGGACCCAAAAATGTCTTGTATTA<br>CAAATATGAGAGCTGGGACATACA
TUSC5	Forward<br>Reverse	CTCAACCTCATCCCCCTCAT<br>CCACCATGATAATGACGATGC
CTAG2	Forward<br>Reverse	CTTCTGCGCAGGATGGAAGGT<br>AGGATCCTGCGGACCAGCTC
CDKN2B	Forward<br>Reverse	CCAACGGAGTCAACCGTTTC<br>GGAGACTCCTGTACAAATCTACATCG
